# Ginsenoside Rh1 Suppresses Vesicular Stomatitis Virus Replication by Inhibiting Autophagy to Promote Immune Responses

**DOI:** 10.3390/microorganisms14040757

**Published:** 2026-03-27

**Authors:** Hongmei Chen, Qinglu Zhao, Dingcheng Wei, Zhanying Hu, Xueliang Zhu, Rui Zhang

**Affiliations:** 1 Key Laboratory of Animal Medicine of Sichuan Education Department, Southwest Minzu University, Chengdu 610041, China; 15775474133@163.com (H.C.); zql3278153307@163.com (Q.Z.); wdc18111950049@163.com (D.W.); 18848260360@163.com (Z.H.); 2 State Key Laboratory for Animal Disease Control and Prevention, Lanzhou Veterinary Research Institute, Chinese Academy of Agricultural Sciences, Xujiaping 1, Yanchangpu, Chengguan District, Lanzhou 730046, China; zhuxueliang@caas.cn

**Keywords:** Ginsenoside Rh1, antiviral, vesicular stomatitis virus, autophagy, immunomodulatory strategy

## Abstract

Vesicular stomatitis virus (VSV), a member of the *Vesiculovirus* genus within the *Rhabdoviridae* family, is a widespread pathogen affecting all hoofed livestock species, leading to reduced animal growth and productivity. To date, no effective therapeutic treatment for VSV infection has been developed. Natural medicinal compounds with immunomodulatory properties represent a promising supportive strategy for infection control. Ginsenoside Rh1, a primary bioactive component of ginseng plants, has been reported to possess broad pharmacological and immunoregulatory activities. Nevertheless, its potential antiviral effects against VSV remain unexplored. In this study, we demonstrate that Ginsenoside Rh1 exhibits considerable antiviral activity against VSV in cellular models. Mechanistically, its antiviral effect is primarily mediated through the inhibition of VSV-induced autophagy, thereby enhancing interferon-mediated antiviral responses. Collectively, our findings identify Ginsenoside Rh1 as a novel antiviral agent active against VSV and potentially related vesiculoviruses, clarify its mechanism of action, and highlight an autophagy-dependent immunomodulatory approach that could be critical for confronting existing and emerging RNA viral infections.

## 1. Introduction

Vesicular stomatitis (VS) is an acute infectious disease that primarily affects hoofed livestock, with characteristic clinical signs including vesicular lesions that are predominantly in the oral mucosa and on the feet [1,2,3]. The disease is often accompanied by fever and may involve a viremic phase. Painful oral lesions can lead to anorexia in affected animals, resulting in weight loss and, in dairy cattle, a decline in milk yield [4]. Transmission to humans is believed to occur primarily through direct contact with infected animals [2,4]. First described following an outbreak in the United States in 1916 [5], VS is currently enzootic in parts of equatorial America, spanning from northern South America to southern Mexico, as well as on Ossabaw Island off the coast of Georgia, USA. Seasonal epizootics, however, extend over a wider geographic range, reaching into the central and southwestern United States and as far north as Canada [6]. While VS is considered primarily a disease of livestock, the detection of neutralizing antibodies in diverse wildlife species—including deer, elk, coyotes, bears, skunks, squirrels, and rats—suggests a broad host range and potential for viral circulation in wild animal populations, even in the absence of overt clinical disease [3,4].

Vesicular stomatitis virus (VSV), a member of the *Vesiculovirus* genus within the *Rhabdoviridae* family, is an important livestock pathogen. Its genome consists of a non-segmented, negative-sense RNA strand approximately 11 kb in length [7]. The genome encodes five structural proteins: nucleoprotein (N), phosphoprotein (P), matrix protein (M), glycoprotein (G), and the large RNA-dependent RNA polymerase (L) [8]. Additionally, two non-structural proteins, C and C’, are expressed via alternative start codons within the P-gene reading frame [9,10]. Genome encapsidation in VSV is driven by specific interactions between the N and P proteins [11]. Beyond its structural role, the N protein is essential for suppressing transcription termination signals during viral replication [12]. The P and L proteins function as co-factors of the viral RNA-dependent RNA polymerase (RdRp), playing multifunctional roles in the initiation, elongation, and encapsidation of viral RNAs [13,14]. The RdRp binds to the encapsidated genome at the leader region and proceeds to transcribe each gene in a sequential, polar manner [15].

Although VSV exhibits broad cellular tropism and efficiently infects a wide range of cell types, its replication is markedly attenuated in cells with a pre-established antiviral state [16]. This finding suggests that VSV intrinsically lacks the ability to directly counteract an active innate immune response upon cellular entry. Therefore, the virus likely depends on hijacking specific host cellular factors to suppress the antiviral environment and enable productive replication [17]. This inherent susceptibility highlights a promising therapeutic approach: boosting the host’s intrinsic antiviral defenses to inhibit VSV replication. Currently, there are no FDA-approved antiviral drugs specifically indicated for VSV infection in livestock, and treatment is primarily supportive [18]. Experimental compounds with reported anti-VSV activity include ribavirin [19,20], 6-azauridine [21], and various natural products such as resveratrol [22] and curcumin [23]. While these compounds have demonstrated efficacy in cell culture models, none have progressed to clinical use for VSV due to concerns about toxicity, bioavailability, or cost.

Ginseng, a widely utilized herbal medicine primarily sourced from Panax ginseng, *Panax quinquefolius* L., and related species, is recognized for its immunomodulatory properties in humans [24,25,26]. Ginsenoside Rh1, one of its notable bioactive constituents, has attracted considerable research interest [26,27,28] and is employed as a quality control marker for *Panax quinquefolius* L., with a specified minimum content of 1.0% [29]. Extracts of ginseng and their metabolites, including Rh1, Rb2, Rg3, and intestinal transformation products such as Compound K [30], have been reported to possess diverse pharmacological activities. These include modulation of immune responses [28,30] and antioxidative and antibacterial effects [31], as well as potential therapeutic benefits in conditions like colitis [32] and diabetes [33]. Fermented ginseng extracts and several ginsenosides have demonstrated a range of antiviral activities. Notably, despite the documented antiviral activities of various ginsenosides against diverse viruses, such as human rhinovirus 3 (HRV3) [34], murine gammaherpes virus 68 (MHV68) [35] and respiratory syncytial virus (RSV) [24,36,37], the antiviral potential of Ginsenoside Rh1 has remained unexplored.

The innate immune system recognizes viral nucleic acids via specialized cytosolic sensors [38]. Among these, retinoic acid-inducible gene I (RIG-I) and melanoma differentiation-associated gene 5 (MDA5) serve as key detectors of viral RNA [39]. Following RNA recognition, activated RIG-I and MDA5 engage the mitochondrial antiviral signaling protein (MAVS). MAVS then recruits and activates TANK-binding kinase 1 (TBK1), which phosphorylates both MAVS and the transcription factor interferon regulatory factor 3 (IRF3). Phosphorylated IRF3 dimerizes and translocates to the nucleus, where it promotes the transcription of type I IFN [38,40]. Secreted IFN-I acts in an autocrine or paracrine manner by binding to cognate cell-surface receptors, thereby triggering the JAK/STAT signaling pathway. This activation leads to the expression of hundreds of interferon-stimulated genes (ISGs), collectively establishing an antiviral state in the host cell [41,42]. Concurrently, signaling through MAVS can also activate the IκB kinase (IKK) complex, resulting in the phosphorylation and degradation of IκB proteins. This process releases transcription factors of the nuclear factor κB (NF-κB) family, allowing NF-κB to enter the nucleus and drive the production of pro-inflammatory cytokines, such as interleukin-6 (IL-6) and tumor necrosis factor-α (TNF-α), thereby amplifying inflammatory responses during viral infection [43,44].

Autophagy, an evolutionarily conserved intracellular lysosomal degradation pathway, plays an essential role in maintaining cellular homeostasis [45,46]. It mediates the removal of damaged organelles, misfolded proteins, and invading microorganisms, thereby protecting cells from metabolic stress, nutrient deprivation, and infection [47,48,49]. Morphologically, autophagy is characterized by the formation of double-membrane vesicles called autophagosomes, which sequester cytoplasmic cargo and deliver it to lysosomes for degradation and recycling. This tightly regulated process consists of three major stages—initiation, elongation, and maturation—coordinated by over 20 conserved autophagy-related (ATG) proteins that assemble at specific endoplasmic reticulum subdomains upon activation. Microtubule-associated protein 1 light chain 3 (LC3) is a widely used molecular marker for monitoring autophagosome formation [50,51]. In addition to its function in cellular quality control, autophagy contributes significantly to both innate and adaptive immunity by participating in host defense against various intracellular pathogens, including bacteria, viruses, and protozoa [52,53]. The mechanisms described for various ginsenosides include direct viral protein targeting [54], modulation of mitochondrial dynamics [55,56], and inhibition of viral enzymes [36,57]. Whether Rh1 possesses antiviral activity and, if so, whether it acts through similar or distinct mechanisms, particularly the role of autophagy, is unknown.

The aim of this study was to evaluate the effect of Ginsenoside Rh1 on VSV replication and to elucidate its underlying mechanism of action in vitro. The findings reveal important molecular pathways influenced by Ginsenoside Rh1, which may inform the development of novel therapeutic strategies against RNA viruses infections.

## 2. Materials and Methods

### 2.1. Cell Culture and Virus Propagation

African green monkey kidney (Vero) cells (ATCC, CCL-81™) were maintained in Dulbecco’s modified Eagle’s medium (DMEM; Gibco, Carlsbad, CA, USA) supplemented with 10% heat-inactivated fetal bovine serum (FBS; Gibco, Carlsbad, CA, USA), 100 U/mL penicillin, and 100 μg/mL streptomycin. Caprine endometrial epithelial (EEC) cells, generously provided by Yongxi Dou (Lanzhou Veterinary Research Institute, Chinese Academy of Agricultural Sciences, Lanzhou, China), were cultured in Dulbecco’s minimal essential medium/Nutrient Mixture F-12 Ham’s medium (DMEM/F12, Carlsbad, CA, USA) containing 10% fetal bovine serum (FBS; Gibco), 100 IU/mL penicillin, and 10 μg/mL streptomycin. Both cell lines were grown as monolayers in cell culture flasks or dishes at 37 °C under a humidified atmosphere of 5% CO_2_.

The VSV stock was obtained from the Lanzhou Veterinary Research Institute, Chinese Academy of Agricultural Sciences. Viral propagation was performed in Vero cell monolayers. Briefly, VSV was inoculated onto Vero cells and cultured in DMEM supplemented with 2% FBS at 37 °C under 5% CO_2_ for five days, until a distinct cytopathic effect (CPE) was observed in approximately 80% of cells. The virus-containing supernatant was collected, and the remaining cells were subjected to three cycles of freezing and thawing for lysis.

Virus titers were determined by inoculating cell monolayers in 96-well plates with 10-fold serial dilutions of viral stock and incubating them at 37 °C for 5–7 days. The 50% tissue culture infective dose (TCID_50_) per milliliter was calculated according to the Reed–Muench method [58]. The multiplicity of infection (MOI) used in each experiment was based on the titer determined for the corresponding cell line.

### 2.2. Antibodies and Reagents

For Western blot analysis, the following primary antibodies were used: anti-GAPDH rabbit mAb (5174), anti-LC3B rabbit mAb (2775), anti–Phospho-IRF3 (Ser396) rabbit mAb (29047), anti-IRF3 rabbit mAb (11904), anti–Phospho-TBK1 (Ser172) rabbit mAb (5483), anti-TBK1 rabbit mAb (3504), anti–Phospho-NF-κB p65 (Ser536) rabbit mAb (3033), anti-NF-κB p65 rabbit mAb (8242), anti-GFP rabbit mAb (2956), anti-ATG16rabbit mAb (8089), and HRP-linked secondary antibodies included anti-rabbit IgG (7074) and anti-mouse IgG (7076) were from Cell Signaling Technology (Danvers, MA, USA). Ginsenoside Rh1 (HY-N0604, purity: 98.73%, formula: 
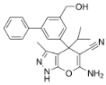
) was obtained from MCE (South Brunswick Township, NJ, USA). Lipofectamine 2000 (11668019) was purchased from Thermo Fisher Scientific (Waltham, MA, USA).

### 2.3. Cytotoxicity Assay

Cytotoxicity was assessed using the Cell Counting Kit-8 (CCK-8) assay (Absin, Shanghai, China, abs50003). Cells were seeded in 96-well plates and cultured for 24 h. The medium was then replaced with 100 µL of fresh medium containing Ginsenoside Rh1 at final concentrations ranging from 0 to 30 µM (0, 10, 20, and 30 µM). Following 48 h of incubation at 37 °C under 5% CO_2_, 10 µL of CCK-8 solution was added to each well, and plates were incubated for an additional 2 h at 37 °C. Absorbance at 450 nm was measured for each sample in quadruplicate using an ELISA microplate reader (PerkinElmer, VICTOR Nivo™, Waltham, MA, USA).

### 2.4. Drug Treatment

Cells were seeded into new culture flasks or dishes. At the time of seeding, Ginsenoside Rh1 was added to the culture medium at a final concentration of 30 µM. After 24 h, cells were either mock-infected or infected with VSV at a multiplicity of infection (MOI) of 1. Following a 1 h adsorption period at 37 °C under 5% CO_2_, the virus inoculum was removed, and cells were washed to eliminate unbound viral particles. Subsequently, the cells were maintained in fresh DMEM supplemented with 2% FBS, with Ginsenoside Rh1 maintained at the indicated concentration in the treated groups. Both cells and corresponding supernatants were collected at 12 hpi for further analysis.

### 2.5. Western Blotting

Cells treated with 30 µM Ginsenoside Rh1 or left untreated were harvested at 12 hpi. After washing with cold PBS, cell lysates were prepared using RIPA buffer containing protease and phosphatase inhibitors. The lysates were clarified by centrifugation, and total protein concentration was determined with a BCA protein assay. Equal amounts of protein were denatured, separated by 10% SDS-PAGE, and transferred onto PVDF membranes. Membranes were blocked with 5% non-fat milk and then incubated overnight at 4 °C with primary antibodies. Following washes, the membranes were incubated with HRP-conjugated secondary antibodies for 1 h at room temperature. Protein bands were visualized using enhanced chemiluminescence (ECL) substrate. GAPDH served as the loading control. Images were captured using a digital imaging system.

### 2.6. Quantitative Real-Time PCR

Total RNA was isolated at 12 hpi from cells treated with 30 µM Ginsenoside Rh1 or from untreated controls using the RNeasy Plus Universal Mini Kit (Qiagen, Hilden, Germany, 73404). First-strand cDNA synthesis was performed using the Maxima H Minus cDNA Synthesis Master Mix (Thermo Scientific, Waltham, MA, USA, M1682). Quantitative real-time PCR (qRT PCR) was conducted with PowerUp SYBR Green Master Mix (Applied Biosystems, Waltham, MA, USA, 1801040) following the manufacturer’s recommended cycling conditions. The primer sequences used were as follows:

VSV forward: 5′-GGCAACATTTGGACACCTCT-3′

VSV reverse: 5′-AAGTGGAAGGCAGGGTTTTT-3′

GAPDH forward (Vero): 5’-CGAGATCCCTCCAAAATCAA-3’

GAPDH reverse (Vero): 5’-TGACGATCTTGAGGCTGTTG-3’

ATG16 forward (EEC): 5’-GGACATGATGGTTCGTGGAA-3’

ATG16 reverse (EEC): 5’-GGTCAATCACCAACTGGGCTA-3’

IFN-β forward (EEC): 5’-TGCCTCCTCCAGATGGTTCT-3’

IFN-β reverse (EEC): 5’-TGACCAATACGGCATCTTCC-3’

ISG56 forward (EEC): 5’-CTGAAATGCGGAGGAAAGAA-3’

ISG56 reverse (EEC): 5’-TCCAGGCGATAGATGACGAT-3’

IL-6 forward (EEC): 5’-TTCCAATCTGGGTTCAATCA-3’

IL-6 reverse (EEC): 5′-TTTCCCTCAAACTCGTTCTG-3′

IL-1β forward (EEC): 5′-ATGCTTCCAATCTGGGTTCA-3′

IL-1β reverse (EEC): 5′-ATGCTTCCAATCTGGGTTCA-3′

GAPDH forward (EEC): 5′-CCACGAGAAGTATAACAACACCC-3′

GAPDH reverse (EEC): 5′-GGTCATAAGTCCCTCCACGAT-3′

### 2.7. RNA Interference

siRNAs targeting the ATG16 gene in EEC cells were synthesized by Sangon Biotech (Shanghai, China). A scrambled siRNA was employed as a negative control. Knockdown efficiency was assessed by quantitative real-time PCR. The siRNA sequences are as follows:

siRNA-ATG16 sense: 5′-GCUGAGAAUUAAACACCAAGA-3′

siRNA-ATG16 antisense: 5′-UUGGUGUUUAAUUCUCAGCUG-3′

For the construction of lentiviral CRISPR-Cas9 vectors, gRNAs are designed using gRNA Designer from Feng Zhang’s lab. Primers are synthesized and cloned into Lenti CRISPR v2 vector by ligation. The target sequence of ATG16 gene is: 5′-ACAGGAAGCGACATGTCGTC-3′. gRNA sequences are as follows:

gRNA1: 5′-AGATGTGCCGCTTCCAGCGG GGG-3′

gRNA2: 5′-GCTGCAGAGACAGGCGTTCG AGG-3′

### 2.8. Lentivirus Packaging and Infection

For lentivirus production, 4 × 10^6^ Lenti X 293T cells (Takara, Beijing, China, 632180) were co transfected with 1.5 µg psPAX2 packaging plasmid (Addgene, Watertown, MA, USA, 12260), 1 µg pMD2.G envelope plasmid (Addgene, 12259), and 2 µg pLKO.1 plasmid using Lipofectamine 2000. The supernatant was harvested at 36 h post transfection, filtered, and stored at −80 °C. Vero cells were transduced with lentiviral particles in the presence of 8 µg/mL polybrene (Solarbio, Beijing, China, H8761) for 24 h, followed by selection with 5 µg/mL puromycin (Invitrogen, Carlsbad, CA, USA, A1113803) for 3 days. Monoclonal Vero cell lines were validated by DNA sequencing. Knockdown efficiency was evaluated by immunoblotting.

### 2.9. Statistical Analysis

Data are presented as mean ± standard deviation (SD). Statistical significance between treatment groups was determined by two-way analysis of variance (ANOVA) using GraphPad Prism software (version 10.0). A *p*-value of less than 0.05 was considered statistically significant.

## 3. Results

### 3.1. Ginsenoside Rh1 Inhibits VSV Replication

**Figure 1:**
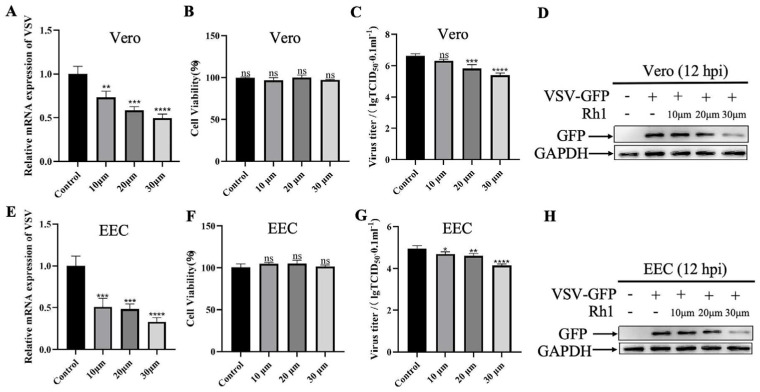
**Ginsenoside Rh1 inhibits VSV replication in a dose-dependent manner.** (**A**) VSV mRNA levels in various concentrations of Ginsenoside Rh1-treated, VSV-infected Vero cells (MOI = 1, 12 hpi) were measured by qPCR. The data show the means ± SD; ** *p* < 0.01; *** *p* < 0.001; **** *p* < 0.0001. (**B**) Cytotoxicity of different concentrations of Ginsenoside Rh1 on Vero cells. Viability was normalized to non-treated control. The data show the means ± SD; n = 3; ns, no significance. (**C**) Virus titers levels in various concentrations of Ginsenoside Rh1-treated, VSV-infected Vero cells (MOI = 1, 12 hpi) were measured by TCID_50_. The data show the means ± SD; *** *p* < 0.001; **** *p* < 0.0001. (**D**) Western blotting analysis of GFP protein in various concentrations of Ginsenoside Rh1-treated, VSV-infected wild-type Vero cells (MOI = 1, 12 hpi). GAPDH was used as a loading control. (**E**) VSV mRNA levels in various concentrations of Ginsenoside Rh1-treated, VSV-infected EEC cells (MOI = 1, 12 hpi) were measured by qPCR. The data show the means ± SD; *** *p* < 0.001; **** *p* < 0.0001. (**F**) Cytotoxicity of different concentrations of Ginsenoside Rh1 on EEC cells. Viability was normalized to non-treated control. The data show the means ± SD; n = 3; ns, no significance. (**G**) Virus titers levels in various concentrations of Ginsenoside Rh1-treated, VSV-infected EEC cells (MOI = 1, 12 hpi) were measured by TCID_50_. The data show the means ± SD; * *p* < 0.05; ** *p* < 0.01; **** *p* < 0.0001. (**H**) Western blotting analysis of GFP protein in various concentrations of Ginsenoside Rh1-treated, VSV-infected wild-type EEC cells (MOI = 1, 12 hpi). GAPDH was used as a loading control.

**Figure 2:**
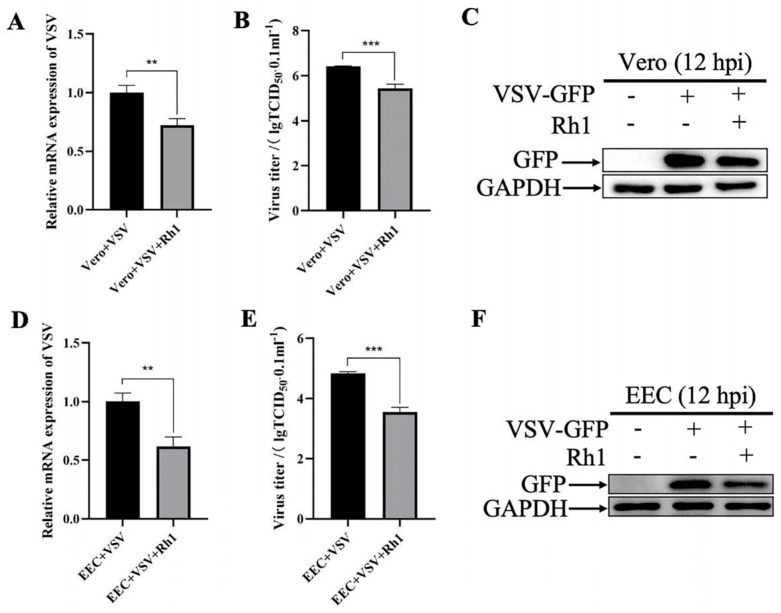
**Ginsenoside Rh1 (30 µM) significantly inhibits VSV replication both in Vero and EEC cells.** (**A**) VSV mRNA levels in 30 µM Ginsenoside Rh1-treated, VSV-infected Vero cells (MOI = 1, 12 hpi) were measured by qPCR. The data show the means ± SD; n = 3; ** *p* < 0.01. (**B**) Control and (30 µM) Ginsenoside Rh1 pre-treated Vero cells were infected with VSV (MOI = 1), and virus titers were measured by TCID_50_ (12 hpi). The data show the mean ± SD; n = 3; *** *p* < 0.001. (**C**) Western blotting analysis of GFP protein in VSV-infected wild-type and Ginsenoside Rh1-treated cells (MOI = 1, 12 hpi). GAPDH was used as a loading control. (**D**) VSV mRNA levels in 30 µM Ginsenoside Rh1-treated, VSV-infected EEC cells (MOI = 1, 12 hpi). The data show the means ± SD; n = 3; ** *p* < 0.01. (**E**) Control and (30 µM) Ginsenoside Rh1 pre-treated EEC cells were infected with VSV (MOI = 1), and virus titers were measured by TCID_50_ (12 hpi). The data show the mean ± SD; n = 3; *** *p* < 0.001. (**F**) VSV GFP protein level in infected wild-type and Ginsenoside Rh1-treated EEC cells (MOI = 1, 12 hpi). GAPDH was used as a loading control.

To examine the effect of Ginsenoside Rh1 on VSV infection, Vero cells were pretreated with increasing concentrations of Ginsenoside Rh1 for 24 h, followed by viral infection and continued compound exposure for 12 h. Quantitative real time PCR analysis performed at 12 hpi showed a dose-dependent reduction in VSV mRNA levels. Although a significant decrease was observed at 10 µM, treatment with 30 µM Ginsenoside Rh1 led to approximately 60% lower viral mRNA compared with the vehicle (DMEM) control (Figure 1A,E), indicating that effective antiviral activity requires relatively high concentrations. Notably, Ginsenoside Rh1 alone did not compromise cell viability (Figure 1B,F), confirming that its inhibitory effect was not due to cytotoxicity. In line with this, viral titers (Figure 1C,G) and viral protein levels (Figure 1D,H) at 12 hpi also declined in a dose-dependent manner with increasing Ginsenoside Rh1 concentrations. Consistent with these findings, pretreatment with 30 µM Ginsenoside Rh1 significantly reduced viral mRNA levels (Figure 2A,D) and infectious virus titers (Figure 2B,E) in both Vero and EEC cells, and markedly decreased viral GFP protein expression as assessed by Western blotting (Figure 2C,F). Collectively, these results indicate that Ginsenoside Rh1 potently suppresses VSV replication in vitro.

### 3.2. Ginsenoside Rh1 Inhibits the Adsorption and Replication of VSV

**Figure 3:**
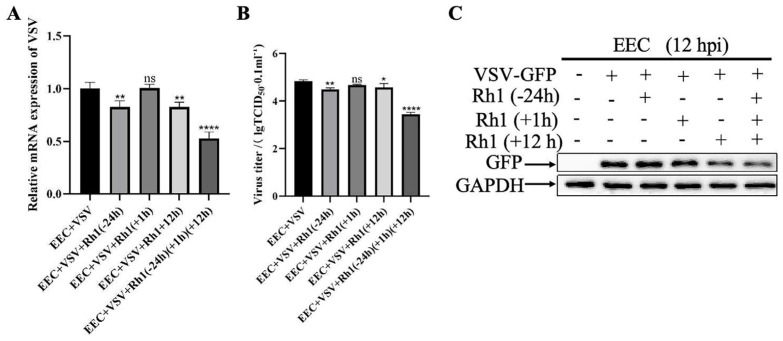
**Ginsenoside Rh1 inhibits the adsorption and replication of VSV.** (**A**) VSV mRNA levels in 30 µM Ginsenoside Rh1-treated, VSV-infected EEC cells (MOI = 1, 12 hpi). The data show the means ± SD; n = 3; ** *p* < 0.01; **** *p* < 0.0001; ns, no significance. (**B**) Control and (30 µM) Ginsenoside Rh1 pre-treated EEC cells were infected with VSV (MOI = 1), and virus titers were measured by TCID_50_ (12 hpi). The data show the mean ± SD; n = 3; * *p* < 0.005; ** *p* < 0.01; **** *p* < 0.0001; ns, no significance. (**C**) Western blotting analysis of GFP protein in VSV-infected wild-type and Ginsenoside Rh1-treated cells (MOI = 1, 12 hpi). GAPDH was used as a loading control.

To investigate the specific stages of the VSV life cycle affected by Ginsenoside Rh1, EEC cells were treated with 30 µM Ginsenoside Rh1 according to four distinct regimens: pretreatment for 24 h before infection, treatment only during the 1 h viral adsorption period, treatment only for 12 h after infection, or treatment both before and after infection. Treatment with Ginsenoside Rh1 before and/or after VSV infection both lead to lower viral mRNA levels (Figure 3A) and infectious virus titers (Figure 3B) than in controls, as determined by RT qPCR and TCID_50_ assays, respectively. Consistent with these results, Western blot analysis showed a marked reduction in VSV GFP protein expression in the presence of Ginsenoside Rh1 before and/or after infection (Figure 3C). The data indicate that Ginsenoside Rh1 suppresses VSV replication dependent on treatment timing. Furthermore, the strongest inhibitory effect was observed when the compound was applied after infection. Collectively, these findings demonstrate that Ginsenoside Rh1 effectively interferes with multiple stages of the VSV life cycle, including attachment and replication, in vitro.

### 3.3. Ginsenoside Rh1 Inhibits VSV-Induced Interferon Responses

**Figure 4:**
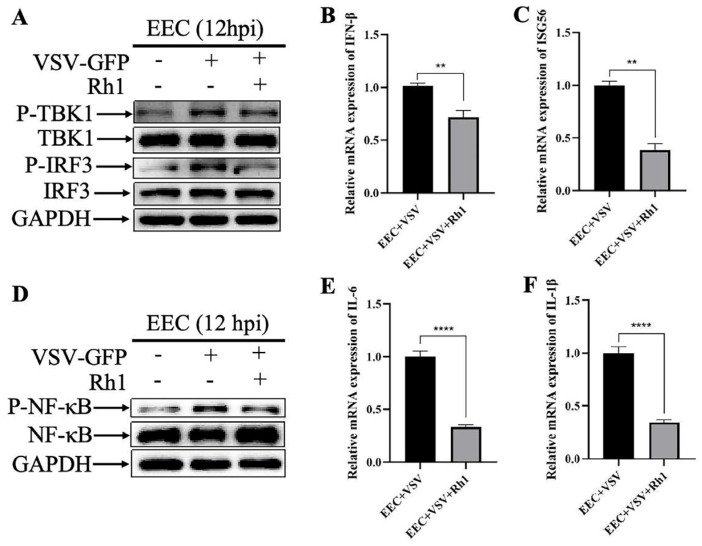
**Ginsenoside Rh1 inhibits VSV-induced immune responses.** (**A**) Western blotting analysis of P-TBK1, TBK1, P-IRF3 and IRF3 protein in VSV-infected wild-type and Ginsenoside Rh1-treated EEC cells (MOI = 1, 12 hpi). GAPDH was used as a loading control. (**B**) IFN-β mRNA levels in 30 µM Ginsenoside Rh1-treated, or untreated, VSV-infected EEC cells (MOI = 1, 12 hpi). The data show the means ± SD; n = 3; ** *p* < 0.01. (**C**) ISG56 mRNA levels in 30 µM Ginsenoside Rh1-treated, or untreated, VSV-infected EEC cells (MOI = 1, 12 hpi). The data show the means ± SD; n = 3; ** *p* < 0.01. (**D**) Western blotting analysis of P-NF-κB and NF-κB protein in VSV-infected wild-type and Ginsenoside Rh1-treated EEC cells (MOI = 1, 12 hpi). GAPDH was used as a loading control. (**E**) IL-6 mRNA levels in 30 µM Ginsenoside Rh1-treated, or untreated, VSV-infected EEC cells (MOI = 1, 12 hpi). The data show the means ± SD; n = 3; **** *p* < 0.0001. (**F**) IL-1β mRNA levels in 30 µM Ginsenoside Rh1-treated, or untreated, VSV-infected EEC cells (MOI = 1, 12 hpi). The data show the means ± SD; n = 3; **** *p* < 0.0001.

To elucidate the mechanism underlying the anti VSV activity of Ginsenoside Rh1, we examined its effects on innate immune signaling in VSV-infected EEC cells. Intriguingly, our findings reveal that Ginsenoside Rh1 suppressed the activation of the TBK1 IRF3 signaling axis induced by VSV infection (Figure 4A), resulting in a significant reduction in the expression of downstream antiviral effectors, including IFN β (Figure 4B) and ISG56 (Figure 4C). Similarly, Ginsenoside Rh1 attenuates the PPRV-triggered activation of the NF κB pathway (Figure 4D) and markedly decreases the production of pro inflammatory cytokines IL 6 (Figure 4E) and IL 1β (Figure 4F). Together, these results demonstrate that Ginsenoside Rh1 inhibits VSV-driven innate immune responses in vitro.

### 3.4. Ginsenoside Rh1 Inhibits VSV-Mediated Autophagy

**Figure 5:**
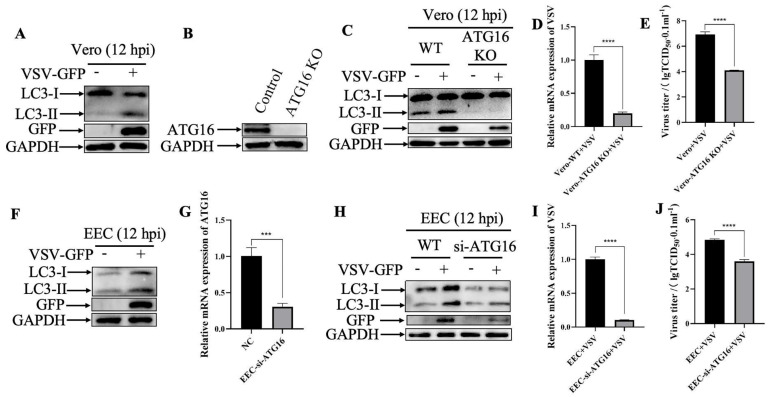
**VSV exploits autophagy to promote its replication.** (**A**) Western blotting analysis of GFP protein and LC3 in VSV-infected wild-type Vero cells (MOI = 1, 12 hpi). (**B**) ATG16 silencing efficiency was verified by Western blotting. GAPDH was used as a loading control. (**C**) Western blotting analysis of VSV GFP protein and LC3 in wild-type and ATG16 KO Vero cells infected with or without VSV infection (MOI = 1, 12 hpi). (**D**) VSV mRNA levels in VSV-infected wild-type and ATG16 KO Vero cells (MOI = 1, 12 hpi) were measured by qPCR. The data show the mean ± SD; n = 3; **** *p* < 0.0001. (**E**) Wild-type and ATG16 KO Vero cells were infected with VSV (MOI = 1) and virus titers were measured by TCID_50_ (12 hpi). The data show the mean ± SD; n = 3; **** *p* < 0.0001. (**F**) Western blotting analysis of GFP protein and LC3 in VSV-infected wild-type EEC cells (MOI = 1, 12 hpi). (**G**) ATG16 mRNA levels in EEC cells transfected with ATG16-siRNA or scrambled siRNA were measured by qPCR. The data show the mean ± SD; n = 3; *** *p* < 0.001. (**H**) Western blotting analysis of GFP protein and LC3 in wild-type and ATG16 KD EEC cells infected with or without GFP infection (MOI = 1, 12 hpi). (**I**) VSV mRNA levels in VSV-infected wild-type and ATG16 KD EEC cells (MOI = 1, 12 hpi) were measured by qPCR. The data show the mean ± SD; n = 3; **** *p* < 0.0001. (**J**) Wild-type and ATG16 KD EEC cells were infected with VSV (MOI = 1), and virus titers were measured by TCID_50_ (12 hpi). The data show the mean ± SD; n = 3; **** *p* < 0.0001.

Autophagy is an evolutionarily conserved intracellular degradation pathway that contributes to cellular homeostasis by catabolizing potentially harmful cytosolic components, including viruses. To explore the involvement of autophagy in the antiviral activity of Ginsenoside Rh1, we first examined autophagic activity in VSV-infected cells by monitoring the conversion of LC3 I to LC3 II, a well-established marker of autophagy. As shown in Figure 5A,F, LC3 II levels were markedly elevated in VSV-infected Vero and EEC cells at 12 hpi compared with uninfected controls, indicating that VSV infection induces autophagy in these cell lines. To further investigate the role of autophagy in VSV replication, we generated ATG16 deficient cells (Figure 5B,G). In these cells, VSV-induced autophagy was substantially suppressed (Figure 5C,H), and autophagic flux was nearly abolished. Notably, loss of ATG16 led to a pronounced decrease in VSV GFP protein expression (Figure 5C,H), viral mRNA levels (Figure 5D,I), and infectious virus titers (Figure 5E,J) compared with infected wild-type cells. Collectively, these results suggest that VSV utilizes the host autophagy pathway to promote its own replication.

**Figure 6:**
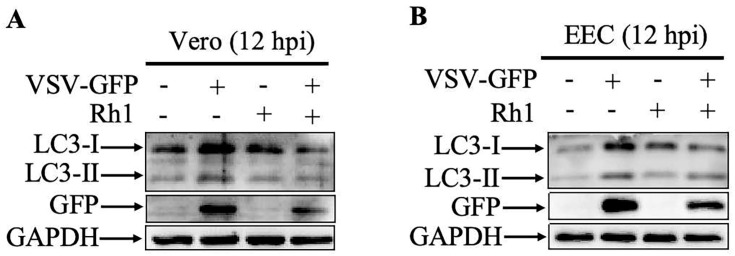
**Ginsenoside Rh1 inhibits PPRV-induced autophagy.** (**A**) Western blotting analysis of GFP protein and LC3 in VSV-infected, Ginsenoside Rh1-treated (30 µM) and untreated wild-type Vero cells (MOI = 1, 12 hpi). (**B**) Western blotting analysis of GFP protein and LC3 in VSV-infected, Ginsenoside Rh1-treated (30 µM) and untreated wild-type EEC cells (MOI = 1, 12 hpi).

To further assess the effect of Ginsenoside Rh1 on VSV-induced autophagy, Vero and EEC cells were infected with VSV at an MOI of 1 for 12 h in the presence or absence of 30 µM Ginsenoside Rh1. The results demonstrate that, while Ginsenoside Rh1 alone did not alter basal autophagy levels, it significantly suppressed the autophagic response triggered by VSV infection in both cell lines (Figure 6A,B). Together, these data suggest that the antiviral activity of Ginsenoside Rh1 against VSV is mediated, at least in part, through the inhibition of virus-induced autophagy.

### 3.5. Inhibition of Autophagy with Ginsenoside Rh1 Promotes VSV-Dependent Interferon Responses

**Figure 7:**
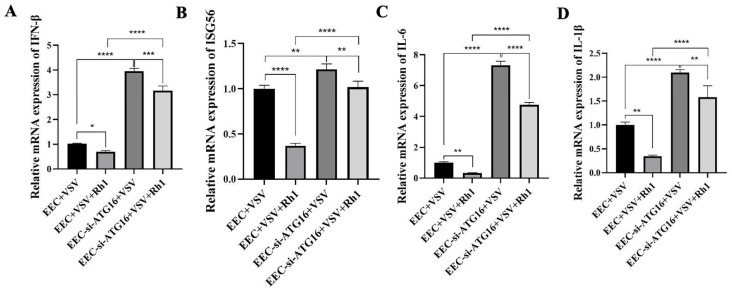
**Ginsenoside Rh1 suppresses VSV-induced autophagy to promote interferon responses.** (**A**) IFN-β mRNA levels in 30 µM Ginsenoside Rh1-treated, or untreated, VSV-infected wild-type and ATG16 KD EEC cells (MOI = 1, 12 hpi). The data show the means ± SD; n = 3; * *p* < 0.005; *** *p* < 0.001; **** *p* < 0.0001. (**B**) ISG56 mRNA levels in 30 µM Ginsenoside Rh1-treated, or untreated, VSV-infected wild-type and ATG16 KD EEC cells (MOI = 1, 12 hpi). The data show the means ± SD; n = 3; ** *p* < 0.01; **** *p* < 0.0001. (**C**) IL-6 mRNA levels in 30 µM Ginsenoside Rh1-treated, or untreated, VSV-infected wild-type and ATG16 KD EEC cells (MOI = 1, 12 hpi). The data show the means ± SD; n = 3; ** *p* < 0.01; **** *p* < 0.0001. (**D**) IL-1β mRNA levels in 30 µM Ginsenoside Rh1-treated, or untreated, VSV-infected wild-type and ATG16 KD EEC cells (MOI = 1, 12 hpi). The data show the means ± SD; n = 3; ** *p* < 0.01; **** *p* < 0.0001.

Given that VSV exploits autophagy to enhance its replication, complete ablation of autophagy (as in ATG16 knockout cells) would be expected to further increase viral yield compared with partial inhibition (as in ATG16 knockdown cells). However, our data showed that VSV GFP protein expression (Figure 5C), viral mRNA levels (Figure 5D), and virus titers (Figure 5E) were all higher in ATG16 knockout Vero cells than in ATG16 knockdown EEC cells (Figure 5H–J). As Vero cells are genetically deficient in interferon production, we hypothesized that autophagy may modulate VSV-induced interferon responses. To test this hypothesis, we measured interferon levels in both wild-type and ATG16 knockdown EEC cells. Compared with PPRV infected wild-type cells, ATG16-deficient cells exhibited significantly elevated mRNA levels of IFN β (Figure 7A), ISG56 (Figure 7B), IL-6 (Figure 7C) and IL-1β (Figure 7D). However, Ginsenoside Rh1 inhibited the production of the above cytokines both in VSV-infected ATG16 knockdown cells and wild-type cells (Figure 7A,B), indicating that the anti-VSV activity of Ginsenoside Rh1 is mainly attributed to its ability to inhibit autophagy, which leads to a promotion of antiviral cytokines production.

## 4. Discussion

Negative-sense RNA viruses employ diverse strategies to counteract the host’s innate immune system [16]. From its initial isolation in 1925 to the clinical approval of the VSV EBOV vaccine in 2019, VSV has undergone a remarkable trajectory—evolving from an agricultural pathogen to a model organism and, ultimately, an established biomedical countermeasure. As a prototype of non-segmented negative strand RNA viruses, VSV has contributed substantially to our understanding of viral replication and host–virus interactions [4]. VSV replicates exclusively in the cytoplasm and expresses five viral proteins, among which the N and M play key roles in subverting host antiviral defenses [59]. During replication, the N protein coats nascent viral genomic RNA, thereby minimizing the formation of double-stranded RNA intermediates and evasion of detection by cytoplasmic RNA sensors that trigger innate immunity [16]. Concurrently, the M protein globally inhibits host gene expression by blocking the nuclear export of cellular mRNAs [59,60,61,62], which attenuates the overall antiviral response during infection. While an effective innate immune response is vital for controlling viral infection, its dysregulation can be detrimental to the host. Therefore, identifying antiviral agents and clarifying the mechanisms through which they modulate these immune pathways are critical for developing strategies to prevent VSV infection.

Ginseng, a medicinal herb derived primarily from the roots of *Panax ginseng* (*Korean/Asian ginseng*), *Panax quinquefolius* (American ginseng), and *Eleutherococcus senticosus* (Siberian ginseng), has long been used in traditional medicine for its immunomodulatory and performance-enhancing properties [37,63]. When administered orally, its major bioactive constituents, ginsenosides, are metabolized by intestinal microbiota into more active derivatives. Fermented ginseng preparations have shown various physiological benefits, including antioxidant and antibacterial effects [30,36], alleviation of colitis [32], and antidiabetic activity [33]. Several ginsenosides exhibit broad-spectrum antiviral activities. For example, 20(R)-ginsenoside Rh2 inhibits gamma herpesvirus replication in both murine and human models [64], while 20(S)-protopanaxtriol shows potent activity against coxsackievirus B3 (CVB3) in vitro [65]. Ginsenosides Re, Rf, and Rg2 protect against rhinovirus and coxsackievirus infections, with Rg2 also displaying significant anti-EV71 activity [66]. Rg3 suppresses hepatitis C virus (HCV)-mediated persistent infection [56], and Rb2 reduces viral titers in models of rotavirus and Sendai virus infection [67,68]. Moreover, Rb2 and Rb3 exhibit antiviral effects against Pestiviruses such as BVDV and CSFV in vitro [69]. Fermented ginseng extracts demonstrate broad inhibition against influenza viruses [36]. Kang et al. have further established Ginsenoside Rb1 as an immunostimulatory agent effective against EV71 in cellular and animal models [24]. While various ginsenosides have been reported to possess antiviral properties against diverse viruses, Ginsenoside Rh1 itself has not previously been shown to have direct antiviral activity. This study provides the first evidence that Ginsenoside Rh1 possesses direct antiviral activity, specifically against VSV. The current study did not include a comparator drug, such as a known antiviral agent or a positive control compound, which would have allowed for a direct assessment of the relative efficacy of Ginsenoside Rh1 against VSV. The absence of such a comparator limits our ability to benchmark the potency of Rh1 against existing or investigational therapies. This is an important limitation that should be addressed in future studies. Future studies should include head-to-head comparisons of Ginsenoside Rh1 with other antiviral compounds, including both natural products (e.g., resveratrol, curcumin) and synthetic agents (e.g., ribavirin, 6-azauridine), under identical experimental conditions. Such comparisons would allow for a more precise assessment of the relative potency, efficacy, and therapeutic index of Ginsenoside Rh1.

Upon pathogen infection, the vertebrate host detects invading agents and initiates precise immune defenses, including the production of IFN. IFN signaling in turn induces hundreds of interferon stimulated genes (ISGs), many of whose encoded proteins directly restrict viral entry, replication, or dissemination, thereby establishing an antiviral state in host cells [70,71,72,73,74]. Ginseng is widely recognized as an herbal tonic with immunomodulatory properties [26]. For instance, Ginsenoside Rb1 enhances both cellular and humoral immune responses in vivo and potentiates IFN signaling, and its antiviral activity is abolished upon knockdown of IFN β [24,69]. Rb1 has also been shown to upregulate IFN α, IFN β, and the interferon-induced protein MxA in specific viral infection models [66,75,76]. Meanwhile, Ginsenoside Rh1 is known to alleviate asthma in mice by restoring Th1/Th2 cytokine balance, reflecting its anti-inflammatory activity [37,77]. Moreover, ginseng extracts reduce the release of pro inflammatory cytokines such as IL 6 and IL 8 while stimulating IFN β expression in influenza challenged mice [78,79]. Similarly, Tao Yu et al. have reported that ginsenoside molecules significantly suppress pro inflammatory cytokines, including TNF α and IL 1 [80]. In this study, we found that Ginsenoside Rh1 significantly inhibits the activation of the TBK1-IRF3 signaling axis and the expression of type I IFN and its downstream antiviral effector ISG56 following VSV infection. Regarding IFN-γ, its expression was below the detection limit in our cell cultures under all conditions tested. The immunomodulatory profile of Ginsenoside Rh1 is distinct from that of other ginsenosides and antiviral agents. While Rb1 enhances IFNβ production to restrict EV71 replication [24] and Rg3 activates IFN I pathway genes in Grass carp reovirus (GCRV) infection [81]. Concurrently, in line with its anti-inflammatory profile, Ginsenoside Rh1 suppresses NF κB activation and reduces the expression of VSV-induced pro inflammatory cytokines. This coordinated suppression of both interferon and pro-inflammatory cytokine responses suggests that Ginsenoside Rh1 may act as a fine tuner of host immunity, potentially preventing excessive or dysregulated immune activation during viral infection. The contrasting outcomes—enhancement of interferon in some models versus inhibition in our VSV system—highlight the context dependency of Ginsenoside actions and underscore the importance of virus- and pathway-specific investigations. The immunomodulatory profile of Ginsenoside Rh1 is distinct from that of other ginsenosides and antiviral agents. While Rb1 enhances IFN-β production to restrict EV71 replication [1] and Rg3 activates IFN-I pathway genes in GCRV infection [5], we were unable to extend this protein-level validation to IFN-β, ISG56, IL-6 and IL-1β due to the lack of commercially available antibodies or ELISA kits validated for goat samples. Overall, these results position Ginsenoside Rh1 as a multifaceted immunomodulator capable of balancing antiviral defense and inflammatory control, offering a promising scaffold for developing targeted host-directed therapeutics against RNA viruses.

Autophagy is an evolutionarily conserved lysosomal degradation pathway that plays a crucial role in maintaining cellular homeostasis and clearing intracellular pathogens [82]. However, many viruses have evolved strategies to hijack this pathway to evade immune detection and enhance their replication. Examples include measles virus (MV) [83], hepatitis C virus (HCV) [84], foot and mouth disease virus (FMDV) [85], influenza A virus (IAV) [86], and dengue virus (DENV) [87]. In line with these observations, our study demonstrates that VSV infection activates autophagy and inhibition of autophagy markedly suppresses viral replication, underscoring the functional importance of autophagy during VSV infection.

Autophagy functions not only as a core cellular defense pathway that eliminates intracellular pathogens via autophagosomal degradation but also plays a key role in initiating innate and adaptive immune responses [52,88,89]. ATG13 has been shown to exert antiviral activity against RNA viruses by promoting IFN production, and depletion of ATG13 significantly impairs RIG I-dependent IFN responses [90]. In contrast, our experiments have demonstrated that the genetic inhibition of autophagy through ATG16 silencing leads to increased expression of both antiviral mediators (IFN β and ISG56) and pro inflammatory cytokines (IL 1β and IL 6). The mechanism we have described—inhibition of virus-induced autophagy leading to enhanced interferon responses—is distinct from those reported for other ginsenosides [91]. Rk1 inhibits IAV by directly binding to the HA1 subunit of hemagglutinin, blocking viral attachment [54]. Rg3 suppresses HCV by restoring Drp1-mediated aberrant mitochondrial fission [56]. Fermented ginseng extracts inhibit both hemagglutination and neuraminidase activity [36]. In contrast, Rh1 acts by modulating a host cellular process (autophagy) to enhance innate immune responses, representing a host-directed rather than virus-directed mechanism. In the present study, we found that Ginsenoside Rh1 inhibits VSV-induced autophagy, thereby suppressing viral replication. Moreover, Ginsenoside Rh1 recapitulated the autophagy inhibitory effect but produced a more refined immunomodulatory profile: it inhibited the expression of antiviral cytokines. The therapeutic implications of IFN suppression versus enhancement are context dependent. For viruses that are highly IFN-sensitive, such as VSV [2], enhancing IFN responses would seem advantageous. However, excessive or dysregulated IFN production can contribute to immunopathology [92,93]. By inhibiting virus-induced autophagy and simultaneously moderating IFN and inflammatory cytokine responses, Ginsenoside Rh1 may achieve a balanced antiviral effect—sufficiently suppressing viral replication while limiting excessive immune activation. This coordinated suppression of both autophagy-driven proviral pathways and host inflammatory signaling positions Ginsenoside Rh1 not merely as an autophagy inhibitor, but as a nuanced immunomodulator capable of fine-tuning the host response to infection, offering therapeutic advantages in balancing antiviral efficacy with reduced immunopathology, particularly in infections where hyperinflammation contributes to disease severity. In this context, Ginsenoside Rh1, as a natural product derived from a widely used herbal medicine, offers potential advantages in terms of safety and tolerability. Ginsenosides have a long history of human use and are generally regarded as safe, which may facilitate their development as veterinary therapeutics.

## 5. Conclusions

In conclusion, this study provides the first demonstration that Ginsenoside Rh1 effectively inhibits VSV replication in vitro. Mechanistic analysis reveals that its anti-viral activity is achieved by blocking VSV-induced autophagy, which in turn promotes antiviral interferon responses. These findings underscore the therapeutic potential of targeting the autophagy pathway as a strategy to counteract RNA virus infection and highlight Ginsenoside Rh1 as a promising candidate for further development as a host-directed antiviral agent.

## Data Availability

The original contributions presented in this study are included in the article. Further inquiries can be directed to the corresponding author.

## Abbreviations

The following abbreviations are used in this manuscript:

VS	Vesicular stomatitis
VSV	Vesicular stomatitis virus
MV	Measles virus
HCV	Hepatitis C virus
RIG-I	Retinoic acid-inducible gene I
MDA5	Melanoma differentiation-associated gene 5
MAVS	Mitochondrial antiviral signaling protein
TBK1	TANK-binding kinase 1
IRF3	Interferon regulatory factor 3
IFN	Interferon
ISG	Interferon-stimulated gene
IKK	IkB kinase
NF-kB	Transcription factors of the nuclear factor κB
IL-6	Interleukin-6
TNF-α	Tumor necrosis factor-α
ATG	autophagy-related gene
LC3	Microtubule-associated protein 1 light chain 3
EV71	Enterovirus 71
FMDV	Foot-and-mouth disease virus
IAV	Influenza A virus
DENV	Dengue virus
GCRV)	Grass carp reovirus
HRV3	Human rhinovirus 3
MHV68	Murine gammaherpes virus 68
RSV	Respiratory syncytial virus
